# H.M. Queen Alexandra's Letter

**Published:** 1918-06-08

**Authors:** 

**Affiliations:** Marlborough House, Pall Mall.


### H.M. Queen Alexandra's Letter.

My Dear Duke,—Many thanks for your kind letter about "my own" Incurable Home at Streatham, in which I have always taken the greatest possible interest ever since I first came to England.

I am truly sorry to hear about its financial position, and most heartily approve of this special appeal of yours on behalf of its funds. I sincerely trust that your efforts to raise £6,000 to meet the deficit may be crowned with success. Alexandra.

Marlborough House, Pall Mall.

